# Adaptation of the G-NORM (Gender norms scale) in Uganda: An examination of how gender norms are associated with reproductive health decision-making

**DOI:** 10.1371/journal.pone.0308249

**Published:** 2024-11-04

**Authors:** Erica Sedlander, Rachel Granovsky, Catherine Birabwa, Dinah Amongin, Ronald Wasswa, Nadia Diamond-Smith, Peter Waiswa, Kelsey Holt, Jeffrey B. Bingenheimer

**Affiliations:** 1 Department of Social and Behavioral Sciences, Institute for Health and Aging, University of California San Francisco, San Francisco, CA, United States of America; 2 Department of Health Policy Planning and Management, Makerere University School of Public Health, Kampala, Uganda; 3 Department of Epidemiology and Biostatistics, University of California San Francisco, San Francisco, CA, United States of America; 4 Department of Family and Community Medicine, University of California San Francisco, San Francisco, CA, United States of America; 5 Department of Prevention and Community Health, George Washington University, Washington, DC, United States of America; University of Nigeria, NIGERIA

## Abstract

**Background:**

Restrictive gender norms exacerbate health inequalities all over the world. More specifically, they prevent women from seeking preventive health services, constrain women’s economic empowerment, and are associated with reproductive health decision making. Gender norms, a subset of social norms, are dynamic and change over time. However, we lack data on how they are changing and how these changes affect health outcomes because current measures do not adequately capture the complex concept of gender norms.

**Methods:**

We originally developed and validated a gender norms scale, the G-NORM, in India. In this study, using cross-sectional data, we adapted the G-NORM from Southeast Asia (India and Nepal) to sub-Saharan Africa (Uganda) in four steps: 1. Formulation of new scale items (via qualitative analysis) 2. Cognitive Interviewing 3. Questionnaire Administration (n = 2422 women of reproductive age) and 4. Psychometric analysis (Confirmatory Factor Analysis).

**Results:**

Like the original scale, descriptive norms and injunctive norms comprised two unique sub scales with high Cronbach’s alphas (.80 & .92). Average scores differed depending on the type of norm suggesting that some gender norms are changing faster than others. Specifically, more equitable injunctive norms were associated with lower odds of partner-dominated contraceptive decision making but descriptive norms were not.

**Conclusions:**

Gender norms serve as a multi-faceted determinant of health and wellbeing and require measurement tools which account for their conceptual complexity. Validating the G-NORM in Uganda expands measurement options for researchers in the sub-Saharan African region working to change norms to reduce health inequalities or to understand the gender normative context before beginning a study.

## Introduction

Restrictive gender norms exacerbate health inequalities all over the world. More specifically, they prevent women from seeking preventive health services [1–3], constrain women’s economic empowerment [4, 5] and are associated with male-dominated reproductive health decision making [6–9]. Gender norms are a subset of social norms that describe how people of a particular gender are expected to behave in a social context. They are embedded within institutions, but they are also dynamic [10] and shaped and understood through communication processes and can thereby be changed to promote healthy behaviors and reduce health inequalities [11, 12]. Global leaders have recognized that gender norms permeate into many facets of women’s lives, and accordingly incorporated gender equity into the United Nations Sustainable Development Goals (SDGs) in 2015. Therefore, there is now unprecedented intent to understand the pathways through which gender norms affect health, with the understanding that conceptually, social norms are a complex phenomenon [10, 13].

Although the term “social norms” is sometimes used loosely as a synonym for individual attitudes, they are quite different and have a strong theoretical basis. They are individual perceptions of *others’* attitudes and behaviors [14]. The widely used Theory of Normative Social Behavior [11, 15] makes the distinction between individual perceptions of what others do, termed *descriptive norms*, and individual perceptions of what others believe one should do, termed *injunctive norms*. Another key element of conceptualizations of social norms is the *reference group*, defined as the set of individuals or groups among whom the norm exists and to whom it applies. This developed conceptual understanding of social norms theory helps us elucidate the pathways through which gender norms affect health outcomes.

### Gaps in gender norms measurement

Despite research showing how restrictive gender norms affect diverse aspects of health and well-being, the processes of developing and validating quantitative measures that adequately capture the phenomena remain in their early stages [16, 17]. Therefore, availability of norms data at scale lags behind global goals to monitor changes in gender norms (e.g., SDG Goal 5 –promote gender equality and empower women) [18].

Given the complexity of social norms, including gender norms, it is not surprising that we lack measures. Specifically, while many researchers measure individual beliefs and behaviors, current scales fail to acknowledge the crucial interdependence between reference group (e.g., friends, family, community) expectations and social action which holds norms in place [19]. For instance, the Gender Equitable Men (GEM) scale measures individual attitudes about general gender roles (i.e. it is a woman’s responsibility to avoid getting pregnant); however, it fails to capture perceptions of normative peer behaviors, which serve to guide our own beliefs and actions [20, 21]. There are two scales [22, 23] that do separate these concepts (among adolescents only,) and one scale that does so for social norms among members of the faith community [24]. To understand the rewards that come with complying with existing norms and the punishments that come from deviating from them, there are calls for quantitative measures to include social sanctions [18].

Responding to these measurement gaps, we originally developed the G-NORM, a gender norms measure in India and then adapted it to Nepal [6, 25]. This paper adapts the scale to a new region—sub-Saharan Africa (Uganda specifically) using a theoretically informed approach to differentiate between descriptive and injunctive norms and capture social sanctions. Like the broader category of social norms, gender norms are context specific and therefore vary across countries, cultures, regions, communities, and institutions. Therefore, gender norms are different in sub-Saharan Africa than in Asia [26]. In a 2023 cross-country study of gender attitudes in low- and middle-income countries using Demographic Health Survey Data, Uganda ranked as one of the most unequal countries in terms of household decision making, contraceptive decision making, and intimate partner violence [27]. Within sub-Saharan Africa, gender norms have been shown to impact a wide range of health outcomes including intention to use contraception, decision making around contraceptive use, fear that contraceptive use may undermine male roles in families, risk of intimate partner violence, and immunization status [2, 7, 24, 28]. Building on this research and given that past G-NORM scales were developed in Southeast Asia, it is critical to have a measure of gender norms that is both theoretically sound and culturally relevant to sub-Saharan Africa. The objective of this study is to adapt and validate the G-NORM scale in a new context (Uganda specifically). To our knowledge, this is the first scale in sub-Saharan Africa that truly captures the overall construct of gender norms, a social phenomenon, among women of reproductive age.

In this adaptation of the G-NORM, we have the following hypotheses:

1. The G-NORM scale in Uganda will fit the same two subfactor structure that we previously identified in India and Nepal (descriptive subscale and injunctive subscale).
2. Just like in India and Nepal, injunctive norms will be higher (more equitable) than descriptive norms.
3. More equitable gender norms will be associated with more equitable reproductive decision making.

## Methods

The data used in this study come from a larger study examining women’s contraceptive decision-making. We used mixed methods from the larger study, to adapt and validate this scale. The qualitative data, in-depth interviews with women of reproductive age, informed the items that we included in the revised scale. The quantitative data come from the baseline wave of a cohort study of sexually active women in two geographically and culturally diverse districts of Uganda–Oyam and Mayuge. Oyam is in Northern Uganda and Maygue is in Eastern Uganda. Both Oyam and Mayuge have a total fertility rate of 7 children per woman and approximately one third of women of reproductive age are using family planning [29]. The qualitative data were collected from February–May 2021 and the quantitative data were collected from January–April 2023.

### Scale development

We adapted and validated the gender norms scale in four stages: (1) formulation of new questionnaire items for the scale based on qualitative data from Oyam and Mayuge Uganda, review of the literature, and expert input; (2) cognitive interviews with draft questionnaire items; (3) determining the dimensionality of the scale and identifying and removing poorly performing items by applying *exploratory factor analysis*; and (4) validation of the scale by applying *confirmatory factor analysis*, and examining associations with outcomes hypothesized to be associated with gender norms.

## Step 1. Formulation of new scale items

### Qualitative data collection and analysis

To ensure that the prior G-NORM items would resonate in the Oyam and Mayuge districts of Uganda and to develop new items, we analyzed qualitative data collected from women of reproductive age in the same districts. See Suchman et al. 2023, for a full description of the qualitative data collection methods [30].

### Qualitative data analysis

- *Coding*. For this study, we conducted a secondary data analysis to examine gender norms in all facets of women’s lives. We used an inductive, thematic approach to code and analyze the interviews, guided by Connells’ Theory of Gender and Power, the Theory of Normative Social Behavior, and the prior G-NORM items [6, 25, 31, 32]. First, two University of California San Francisco, (UCSF) researchers reviewed 20 transcripts (40 total) using open coding to develop an initial list of descriptive codes. Researchers also wrote an analytic memo for each transcript, detailing respondent characteristics and key takeaways. Next, researchers coded the transcripts using Dedoose software.
- *Thematic analysis*. Once transcripts were coded, researchers conducted thematic analysis, distilling additional G-NORM items by reviewing the content of coded data excerpts for common themes. For instance, many segments were double coded with “violence and punishment” and “family planning decision making” leading to G-NORM item, “In most families you know, if a woman uses family planning without permission and her husband (partner) finds out, it leads to violence.” Through this thematic analysis, researchers formulated twelve additional gender norm items.
- *Qualitative findings*. Participant demographics from the subset of sixteen in-depth interviews mirrored those of the original 60 in-depth interview sample. All items from the previous G-NORM scale remained relevant and findings supported additional G-NORM items unique to Uganda.
- *Item development based on qualitative findings*. UCSF researchers then shared the new Uganda G-NORM items and prior G-NORM items with two Makerere School of Public Health (MakSPH) researchers from Uganda for review. The team worked together to isolate the most salient themes and determine which items from the previous India and Nepali scales remained relevant, leaving 22 draft items (10 from the Nepali scale + 12 additional).

Like in prior G-NORM scales, to improve clarity, all questions were written in the same direction to represent inequitable norms and to capture community level gender norms, all questions referred to a single referent group (the community, written as “in most families you know.”) [33]. Finally, to ensure face validity and content validity, we shared all items with three experts in gender norms both from the United States and Uganda. Next, MakSPH researchers translated items from English into the local languages (Lusoga and Langi).

## Step 2. Cognitive interviewing

Following cognitive interview training, two research assistants from Uganda conducted ten cognitive interviews (five in each district). Interviews were conducted in person and consisted of interviewers asking participants to respond to scale items one at a time, reflect on how they came to their conclusion, whether the item was clear or confusing, and if so, why. The cognitive interviews were recorded, and interviewers captured responses via tablets. Cognitive interviews were then translated and transcribed to English. Next, four researchers from the U.S. and Uganda met to review areas of confusion and potential revisions.

### Cognitive interview results

Based upon cognitive interview results, we dropped five items. For example, we dropped the item, (“Most families you know believe that women should eat after everyone has been served”) due to lack of relevance, as most respondents noted that this was not a common practice. We also added a sentence in the directions stating that we are asking about perceptions about what other families are doing, not what the woman herself is doing, and this is not always easy to know, especially for private behaviors, so it is just their best guess.

## Step 3. Questionnaire administration

### Inclusion criteria

As previously mentioned, we added G-NORM items to a survey that was examining women’s contraceptive decision-making. Therefore, women were eligible for the survey if they were between the ages of 15–45 years old, were currently sexually active, not currently pregnant, and as this is the first wave of a longitudinal study, would remain in the study area for the next year. Participants who were new users of a contraceptive method or were not using a contraception were oversampled due to the objectives of the larger study. Research assistants consented participants and then read questions out loud from a tablet and marked down responses using ODK software. Participants were compensated 30,000 Ugandan Shillings (about $8 U.S. dollars).

## Step 4. Psychometric evaluation

To assess the psychometric characteristics of the scale, we employed the same methods we used to modify the original G-NORM scale’s adaptation from India to Nepal [6] (Refer to Sedlander et al. 2023). Initially, we assessed the range, mean, and standard deviation of each item [33]. Subsequently, following the model outlined by Vu et al. 2017 [34] and considering the inclusion of novel items reflecting the Ugandan context, we opted to start with exploratory factor analysis (EFA) [34]. This allowed us to examine the scale’s dimensionality and to eliminate poorly performing items. We constructed a scree plot using eigenvalues to determine the factor count, retaining factors with eigenvalues of 1 or higher. We also visually inspected the scree plots to confirm the accurate extraction of factors. These analyses indicated a two-factor solution. Subsequently, we conducted EFA again but this time constraining the factor count to two. We did not assume the factors would be independent, so we implemented an oblique promax rotation and obtained standardized factor loadings from this solution. Next, we assessed the loadings on each factor and sequentially removed items with factor loadings below 0.4, starting with the least loaded item and progressing to the next lowest, and so on until all remaining items possessed factor loadings of 0.4 or higher [35] (Table 2). Analogous to prior adaptations, we stipulated that items must be eliminated in pairs (e.g., if a certain injunctive norm item is excluded, the corresponding descriptive norm item must also be removed, and vice versa). The rationale for this, explained in detail in our prior scale validation in India (Refer to (Sedlander et al. 2022) [25], aligns with the Theory of Normative Social Behavior where differentiation between descriptive and injunctive norms is pivotal [11, 15]. As EFA serves as a data reduction technique, this approach created a more concise scale. Table 2 illustrates the initial pool of all 30 items with their corresponding factor loadings for each subscale, along with the final items that remained.

Next, we conducted three additional sets of analyses to validate the G-NORM in this new context. First, we conducted confirmatory factor analyses (CFA) on the same sample to evaluate the fit of the two-factor model. In these analyses, we first imposed the assumption of conditional independence, that is, we did not let any item-level uniquenesses correlate with each other. We then relaxed this assumption by allowing the errors/uniquenesses of analogous descriptive norms and injunctive norms items to be correlated. Thus, we compared two, two-factor models: (1) no correlated errors; (2) error correlations between analogous pairs of items. To examine model fit, we used the model chi-squared, the Bentler Comparative Fit Index (CFI), the Tucker–Lewis Index (TLI), the Root Mean Square Error of Approximation (RMSEA), Standard Root Mean Square Residual (SRMR), Aikaike Information Criteria (AIC), and Bayesian Information Criteria (BIC). We conducted all analyses in Stata 18.0. Good-fitting models are indicated by a Tucker-Lewis (TLI) and Comparative Fit Index (CFI) equal to or greater than .90 and a Root Mean Square Error Approximation (RMSEA) less than .08, and standardized root mean squared residual (SRMR) less than .10. Aikake Information Criteria (AIC) and Bayesian Information Criteria (BIC) are interpreted as smaller numbers signify a better fitting model [36].

Second, to provide additional evidence for the validity of the scale, and because gender norms have been shown to be associated with sexual and reproductive health decision making [6] (Sedlander et al. 2023), we used regression models to test if the G-NORM would be associated with three items from the larger study. The first item asked about agreement with the following statement, “there will be conflict in my marriage if I use contraception” (coded using the original categorical responses from strongly agree to strongly disagree (score range from 4 to 1 with higher scores agreeing that using contraception would cause conflict in their marriage). Next, we looked at the association of the G-NORM and agreement with the statement, “If I use family planning my husband/partner may seek another sexual partner.” We used the same categorical responses from strongly agree to strongly disagree (score range from 4 to 1 with higher responses meaning more agreement that using contraception would make your husband/partner seek another sexual partner). We then looked at the association of the G-NORM with women’s responses to a question about if there was a disagreement about using contraception, who would make the final decision (“If there is a disagreement, my partner makes the final decision about whether I use contraception).” We recoded this as a binary response (1 = husband or partner decides and 0 if it was a joint decision or her decision alone). Next, we examined if these items were associated with the G-NORM in the hypothesized direction. To do so, we ran linear or logistic regression models controlling for age (continuous), education (categorical: never went to school or less than primary school, primary school, secondary school, higher than secondary school), religion (catholic as compared to Muslim, Other, Pentecostal and Protestant), age started living with partner (a proxy for age at marriage) (continuous), number of children (continuous), whether the family has a bank account (binary), whether the family owns a mobile phone (dichotomous), husband’s age (continuous), and husbands education (categorical: never went to school or less than primary school, primary school, secondary school, higher than secondary school). We selected these covariates based on previous literature suggesting they are associated with gender norms and reproductive agency [25, 37].

Lastly, because we found in India and Nepal that more educated women held more equitable gender norms, we examined education differentials in the average descriptive and injunctive subscale scores for both women and their husbands. Throughout the analyses, we reversed scored the items to improve interpretability; here higher G-NORM scores correspond to more equitable gender norms.

### Ethical considerations

This study has been approved by the University of California, San Francisco Institutional Review Board as well as the Makerere University School of Public Health Research and Ethics Committee and the Uganda National Council of Science and Technology. Written informed consent was obtained from all participants, they offered a copy of the consent form to take home if they wanted, and their confidentiality was ensured by using anonymous ID numbers for data analysis. Participants ages 15–17 were emancipated minors in Uganda context meaning that they completed informed consent on their own like the participants 18 years and older.

## Results

### Descriptive statistics

**Table 1** provides a description of the 2,422 sexually active women aged 15–45 whose survey data contributed to the psychometric analysis.

**Table 1:** Description of the sample (N = 2,422).

Age Mean (SE)	
	26.5 (.13)
**Education (%)**	
Never went to school or less than primary school	131 (5.4%)
Primary school	1,642 (67.8%)
Secondary school	550 (22.7%)
Higher than secondary school	99 (4.09%)
**Religion**	
Catholic	822 (33.9%)
Muslim	507 (20.9%)
Protestant	712 (29.4%)
Pentecostal	341 (14.1%)
Other	40 (1.6%)
**Partner Status**	
Currently married	1,933 (79.81%)
Partner/boyfriend	428 (17.67%)
Not currently in union: divorced/separated/widow included	40 (1.65%)
Never in union	21(0.87%)
**Age started living with partner** Mean (SD)	18.86 (.07%)
**Has children**	
Yes	2,347 (99.03%)
No	23 (0.97%)
**Number of children** Mean (SD)	2.82 (.037)
**Currently using contraception**	
Yes	2,001 (82.62%)
No	421 (17.38%)
**Someone in the household owns a mobile phone**	
Yes	2,215 (91.45%)
No	206 (8.51%)
**Someone in the family has a bank account**	
Yes	2,085 (86.09%)
No	336 (13.87%)
**Husband age Mean (SD)**	32.2 (.17)
**Husband Education**	
Never went to school or less than primary school	54 (2.29%)
Primary school	1,194 (50.57%)
Secondary school	804 (34.05%)
Higher than secondary school	221 (9.36%)
Don’t know	88 (3.73%)

### Initial psychometric analysis

We reviewed scree plots from exploratory factor analyses for all 30 gender norms items, along with the application of the eigenvalue > 1 rule, suggesting a two-factor solution (factor 1 = descriptive norms and factor 2 = injunctive norms). Factor loadings from the two-factor solution for all 30 items are presented in **Table 2**. As shown in the first two columns, almost all descriptive norms items loaded onto factor one and almost all injunctive norms items loaded onto factor two. Five items, three injunctive norms and two descriptive norms items, had factor loadings of 0.40 or below, so we removed them and their mirrored pair (n = 10). The remaining 20 items are shown in the third and fourth columns of **Table 2**, all of which loaded above 0.40. Both factors have high Cronbach’s alpha scores–a measure of internal consistency (0.80–descriptive norms, 0.92–injunctive norms), meaning that the set of items are closely related to each other. The final column includes the mean score for each item. As noted, injunctive norms (perceived expectations), were more equitable than descriptive norms (perceptions about what is happening in their community).

**Table 2:** Exploratory factor loadings for the Uganda G-NORM scale specifying two factors.

Items	Original Items Factor 1	Original Items Factor 2	Reduced items that loaded above .4 –Factor 1	Reduced items that loaded above .4 –Factor 2	Mean score higher scores = more agreement with the statements
**Descriptive norms: “In most families you know…”**					
1. girls get married before they are 18 years old, but boys wait until they are older.	0.11	0.32			2.60
2. women obey their husbands in all matters.	-0.07	0.42			2.67
3. women ask permission from their husband to leave the house.	-0.00	0.46			2.75
*4. if a woman does not consult her husband about important decisions, it leads to violence.	-0.07	0.37			2.87
*5. if a woman earns money, it will cause problems in her marriage.	-0.13	0.43	-0.13	0.45	2.52
6. only men make decisions about household income and expenses.	0.08	0.61	0.06	0.65	2.49
7. husbands make the final decision about how many children to have.	0.13	0.51	0.130	0.53	2.55
8. men make the final decision about their wife (or partner) using family planning methods.	0.16	0.50	0.160	0.50	2.50
*9. if a woman uses family planning without permission and her husband (partner) finds out, it leads to violence.	0.00	0.39			2.80
*10. if a woman disobeys her husband, she is sent back to her parents (or sent away).	-0.00	0.53	0.03	0.45	2.69
11. only women do the cooking, cleaning, and caring of children.	-0.02	0.53	-0.01	0.55	2.80
12. women stop working when they get married.	-0.11	0.56	-0.13	0.62	2.14
13. girls stop going to school if they get pregnant.	-0.02	0.41	-0.03	0.43	2.80
14. husbands make the final decisions about buying major household items (e.g., television, bicycle, cell phone)	0.02	0.55	0.03	0.56	2.68
15. if there is only enough money for one cell phone for the household, the husband owns it.	0.05	0.52	0.05	0.56	2.72
**Injunctive norms: “Most families you know believe that…”**					
16. girls can get married before they are 18 years old, but boys should wait until they are older.	0.78	-0.07			1.88
17. women should obey their husbands in all matters.	0.02	0.36			2.85
18. women should ask permission from their husband to leave the house.	-0.01	0.43			2.88
19. it is acceptable for a man to respond violently if his wife does not consult him about important decisions	0.79	-0.01			1.88
20. a woman should not work outside the home to keep peace in her marriage.	0.74	0.03	0.72	0.03	1.88
21. only men should make decisions about income and expenses.	0.80	0.04	0.82	0.02	2.03
22. husbands should make the final decision about how many children to have.	0.82	0.00	0.84	-0.03	2.14
23. men should make the final decision about their wife using family planning.	0.82	-0.01	0.83	-0.04	2.06
24. it is acceptable for a husband to respond violently if his wife uses family planning without his permission.	0.87	-0.04			1.78
*25. if a woman disobeys her husband, she should be sent back to her parents (or sent away).	0.61	0.09	0.65	0.01	2.26
26. only women should do the cooking, cleaning, and caring of children.	0.56	0.15	0.61	0.10	2.50
27. women should stop working when they get married.	0.72	0.05	0.69	0.08	1.54
28. girls should stop going to school if they get pregnant.	0.77	-0.08	0.76	-0.09	2.09
29. husbands should make the final decisions about buying major household items (e.g., television, bicycle, cell phone).	0.65	0.09	0.69	0.05	2.33
30. if there is only enough money for one cell phone for the household, the husband should own it.	0.57	0.15	0.60	0.12	2.40

Notes: All response options are on a 4-point likert scale: strongly disagree, disagree, agree, strongly agree. Uganda specific items are highlighted in grey. Items that indicate social sanctions (i.e., repercussions if someone breaks a social norm) have an asterik *

*Descriptive norms & injunctive norms are mirrored pairs. When one item did not load well, we removed the pair.

**Table 3** shows the final gender norms items that we retained after all analysis. More than half of the items (12) are new items based on this adaptation in Uganda.

**Table 3:** Uganda G-NORM scale (20 items total–ten items in each sub-scale).

**Descriptive norms**
*In most families you know if a woman earns money, it will cause problems in her marriage.
In most families you know only men make decisions about household income and expenses.
In most families you know husbands make the final decision about how many children to have.
In most families you know men make the final decision about their wife (or partner) using family planning methods.
*In most families you know if a woman disobeys her husband, she is sent back to her parents (or sent away).
In most families you know only women do the cooking, cleaning, and caring of children.
In most families you know women stop working when they get married.
In most families you know girls stop going to school if they get pregnant.
In most families you know husbands make the final decisions about buying major household items (e.g., television, bicycle, cell phone)
In most families you know if there is only enough money for one cell phone for the household, the husband owns it.
**Injunctive norms**
Most families you know believe that a woman should not work outside the home to keep peace in her marriage.
Most families you know believe that only men should make decisions about income and expenses.
Most families you know believe that husbands should make the final decision about how many children to have.
Most families you know believe that men should make the final decision about their wife using family planning
Most families you know believe that if a woman disobeys her husband, she should be sent back to her parents (or sent away).
Most families you know believe that only women should do the cooking, cleaning, and caring of children.
Most families you know believe that women should stop working when they get married.
Most families you know believe that girls should stop going to school if they get pregnant.
Most families you know believe that husbands should make the final decisions about buying major household items (e.g., television, bicycle, cell phone).
Most families you know believe that if there is only enough money for one cell phone for the household, the husband should own it.

Notes: All response options are on a 4-point likert scale: strongly disagree, disagree, agree, strongly agree

Uganda specific items are highlighted in grey. Items that indicate social sanctions (i.e., repercussions if someone breaks a social norm) have an asterik next to them *

### Confirmatory factor analyses

**Table 4** shows model fit statistics from two confirmatory factor analysis models. Model 2, which includes pairwise correlations among analogous descriptive and injunctive norms, presents the best fit. And all fit statistics in Model 2 indicate a good fitting model.

**Table 4:** Model fit statistics from two confirmatory factor analysis models (n = 2,415).

	Two factor (descriptive and injunctive) norm model	
Factor Structure	Model 1	Model 2
Correlated Errors	None	Analogous Pairs
Fit Statistics		
RMSEA	0.101	0.068
CFI	0.819	0.922
TLI	0.796	0.907
SRMR	0.100	0.050
Chi-squared	4296.284 model vs. saturated 22980.067 baseline vs. saturated	1938.590 model vs. saturated 22980.067 baseline vs. saturated
AIC	111956.641	109618.947
BIC	112309.798	110029.999

Notes; Model two shows the final and best fitting model. Good-fitting models are indicated by a Tucker-Lewis (TLI) and Comparative Fit Index (CFI) equal to or greater than .90 and a Root Mean Square Error Approximation (RMSEA) less than .08, and standardized root mean squared residual (SRMR) less than .10. Aikake Information Criteria (AIC), Bayesian Information Criteria (BIC)–smaller numbers = better fitting model (Vandenberg and Lance, 2000).

### Associations between the G-NORM and reproductive health attitudes and decision making

To examine construct validity, we measured the association between injunctive and descriptive norms and three statements representing different aspects of reproductive attitudes and decision making. Among married women, 22 percent of women strongly agreed or agreed that using contraception would cause conflict in their marriage. Similarly, 22 percent of women agreed or strongly agreed that if they use family planning, their husband/partner may seek another sexual partner. And along the same lines, almost a fifth (19.5 percent) reported that if there is a disagreement, their partner makes the final decision about whether they use contraception.

First, we ran separate models for each subscale independently, and then a model for each outcome with both the descriptive and injunctive norms subscales together. **Table 5** shows that injunctive norms were significantly associated with all three items, both individually and when both were included in the same model; however, descriptive norms were not. Specifically, higher (more equitable) injunctive gender norms were negatively associated with agreeing that, “There will be conflict in my marriage if I use contraception” in the individual model (b = -.075, 95 percent CI -.12 -.020) and when we included descriptive and injunctive norms in the same model (b = -.082, 95 percent CI -.14 -.02)). Similarly, more equitable injunctive gender norms were negatively associated with agreeing that, “If I use family planning my husband/partner may seek another sexual partner,” (b = -.10, 95 percent CI -.16 -.04) in the individual model and when we included descriptive and injunctive norms in the same model (b = -.11, 95 percent CI -.17 -.05). Having more equitable injunctive gender norms was also associated with lower odds that “If there is a disagreement, my partner makes the final decision about whether I use contraception” in the individual model (b = .58, 95 percent CI .45 - .75) and when we included both subscales in the same model (b = .58, 95 percent CI .45–1.59). The association between injunctive norms and each outcome changed negligibly when descriptive norms were included.

**Table 5:** Associations between the G-NORM scale and contraceptive attitudes and decision making.

	There will be conflict in my marriage if I use contraception (regression) n = 2200		If I use family planning my husband/partner may seek another sexual partner (regression) n = 2199		If there is a disagreement, my partner makes the final decision about whether I use contraception (odds ratio) n = 1,254	
	Each subscale separately	Both subscales in the same model	Each subscale separately	Both subscales in the same model	Each subscale separately	Both subscales in the same model
**Descriptive Norms**	-.01 (CI -.11–.07)	.032 (CI -.06–13)	-.02 (CI -.12–06)	.04 (CI -.05–.15)	.76 (CI:.50–1.16)	1.01 (CI.64–1.59)
**Injunctive Norms**	-.075** (CI -.12 -.020)	-.082** (CI -.14 -.02)	-.10*** (CI -.16 -.04)	-.11*** (CI-.17 -.05)	.58*** (CI:.45 - .75)	.58*** (CI.45 - 1.59)

Notes: All response options are on a four-point likert scale from 1–4. Higher scores = more equitable gender norms. We controlled for the following: age, education, religion, whether the family has a bank account, household phone ownership, number of children, husband’s age, husband’s education, age started living with their partner. Confidence intervals in parentheses.

*** p<0.001

** p<0.01

* p<0.05

### Gender norms mean and standard errors and breakdown by educational attainment

**Table 6** shows that reported descriptive gender norms were slightly lower (or less equitable) than injunctive gender norms in the overall sample (descriptive norms = 1.40, CI: 1.38–1.42) and (injunctive norms = 1.87, CI: 1.83–1.90). In other words, women were reporting more equitable injunctive gender norms (expectations) than *actions (actual behavior)*. The difference held across different levels of education for women and their partners. We also show that more educated women and their partners have more equitable gender norms.

**Table 6:** Mean and confidence interval of the G-NORM and breakdown by level of education of participants and their partner.

	Descriptive Norms	Injunctive Norms
	Mean (CI)	Mean (CI)
**Overall Sample**	1.40 (CI: 1.38–1.42)	1.87 (CI: 1.83–1.90)
**Education**		
Primary school or less	1.33 (CI: 1.31–1.35)	1.63 (CI: 1.59–1.67)
Secondary school or higher	1.60 (CI: 1.56–1.64)	2.52 (CI: 2.44–2.59)
**Partner Education**		
Primary school	1.32 (CI: 1.30–1.34)	1.60 (CI: 1.56–1.64)
Secondary school or higher	1.50 (CI: 1.47–1.53	2.15 (CI: 2.10–2.21)

Notes: Gender norms are on a 4-point likert scale: strongly disagree, disagree, agree, strongly agree (higher numbers = more equitable gender norms)

Lastly, we found that the two subscales, descriptive and injunctive gender norms, were modestly and significantly correlated (0.46 p < .001).

## Discussion

This study used a mixed-methods approach to adapt and validate a gender norms scale originally developed in India, and then adapted to Nepal, to a new setting: Uganda. Our findings confirmed that as hypothesized, the two-factor model (descriptive norms and injunctive norms) comprising 10 items each did indeed fall onto two separate factors and effectively represent the overall construct of gender norms. After adding culturally relevant items to the Ugandan context, the final scale resulted in twelve new items compared to prior scales. Both subscales had high inter-item correlation, and confirmatory factor analysis showed that all fit statistics corresponded to a good fitting model. As hypothesized (and like in India and Nepal), injunctive norms were more equitable than descriptive norms. Our third hypothesis was only partially confirmed as injunctive norms were statistically associated with decision making around contraceptive use, but descriptive norms were not.

Our discovery that the two-factor scale remained consistent, contrasted with the experience of the GEM scale during its validation across various settings. Originally developed with two factors (subscales) in Brazil, the GEM scale was validated in Uganda and India. However, in these new countries, the anticipated two-factor structure did not hold. Instead, a single overarching factor was used [20, 34, 38]. Fleming et al. 2018 found that when they used two factors, items loaded poorly or double-loaded, even when attempting to drop problematic items [38]. A similar scenario was observed by Fitriana et al. 2022 when they adapted the “Happiness at Work” scale to the Indonesian context [39]. The scale’s overall structure did not maintain across countries. Authors posited that this could be due to cultural differences such as the collectivist culture in Indonesia, which could influence point of views around happiness at work. These findings strengthen the argument to adapt and validate scales to each context to ensure that researchers are measuring what they intend to. Like our findings, a 2021 study with adolescents in the Democratic Republic of Congo, found that gender norms were weakly associated with overall agency and decision making; suggesting that norms and decision making are unique but related concepts [40]. Our prior work in Nepal along with others also corroborates our findings that sexual and reproductive decision making is associated with gender norms [6, 37, 24]. We also note that as the sexual and reproductive health field moves towards measuring change in contraceptive agency rather than change in contraceptive use, we intentionally chose equity-based outcomes around decision-making rather than simply contraceptive use [41–43].

The main contribution of this paper is providing a validated scale to researchers and interventionists working in sub-Saharan Africa either on gender norms or other gender norms-related programs. Given that restrictive gender norms permeate into almost all aspects of women’s lives worldwide, yet are often not accurately measured, researchers across fields could benefit from a scale that is both conceptually and empirically sound. This tool, now adapted to the sub-Saharan African context, can illuminate not only how much specific gender norms change over time but how much this change contributes to changes in health inequalities. The new items that we added cover women’s economic empowerment including educational opportunities, access to digital tools, and include social sanctions for not complying with these norms. Interventions targeting these aspects of gender empowerment now have a tool to understand the gender normative context before beginning an intervention and testing how they change over time. These new items are both specific to the Ugandan context but also broad enough to cover gender norms across sub-Saharan Africa.

We were surprised to find that descriptive norms were not associated with contraceptive attitudes and decision making as predicted. One potential explanation may be that the descriptive norms subscale was associated with different demographic variables than the injunctive subscale. Indeed, while the subscales were significantly correlated (.46 p < .001), they were also conceptually and as indicated during factor analysis and regression analysis, empirically quite distinct. Another factor that may partially explain this finding is that the injunctive norms subscale had higher internal consistency than the descriptive norms subscale, so its association with outcomes would be less attenuated by measurement error. On the other hand, both subscales had satisfactory internal consistency, making it unlikely that measurement error played a substantial role here. Whatever the explanation, this finding highlights the importance of measuring these concepts separately. Therefore, if practitioners have the resources, we encourage using both sub-scales to measure gender norms. Given that injunctive norms are more equitable than descriptive norms, this suggests that *injunctive norms (perceptions about expectations) may change faster than descriptive norms (perceptions about actual behavior)*. Therefore, specifically targeting injunctive norms may be a logical first step for an intervention attempting to change norms to reduce health inequities. For example, messaging for a women’s economic empowerment intervention could state that “*most husbands believe men and women should make decisions about income and expenses together*.” This is not stating that most do but that’s the expectation (injunctive norm). Subsequently, as this gender norm becomes more equitable over the course of the intervention—and men and women are indeed making more financial decisions together–messaging that “*most couples in this community make financial decisions together*” (descriptive norms) could influence actual behavior.

To adequately measure gender norms and how they change over time, it is critical to measure them on a population level. However, population-based surveys (e.g., the Demographic Health Survey—DHS and Performance Monitoring for Action–PMA) currently do not include gender norms. At the time of writing this, these surveys only include individual attitudes and behaviors related to gender and are critically missing perceptions from the reference groups (the social part of social norms). Additionally, while this scale crosses continents from Asia to Africa to provide a sub-Saharan African scale, other continents like Europe and North and South America have different gender norms. Adapting the G-NORM scale to other regions and contexts (including urban contexts) is a logical next step. We note that this process is resource intensive, so we recommend conducting cognitive testing with the G-NORM scale that is most culturally relevant to the context. Furthermore, vetting items with local gender norm experts, will help ensure that items are capturing gender norms in a new context.

### Limitations

One limitation of this study is that data collection only took place in two rural districts within Uganda. Therefore, these findings may not be representative of urban Ugandan women, Ugandan women as a whole, or women in all of sub-Saharan Africa. Additionally, this sample only includes women. Future research should examine gender norms among men. Furthermore, due to the nature of the larger study, the population was a convenience sample, over sampled to include primarily new users of contraception, so it is not representative of all women of reproductive age in these regions. However, the final sample was diverse with representation from key socio-demographic characteristics.

Additionally, while the overall G-NORM scale comprises myriad dimensions of gender norms, including women’s economic empowerment, time use, digital access, and reproductive health decision making, we were only able to demonstrate construct validity using contraceptive decision making, because the parent study focused on reproductive health. If the larger study had focused on gender norms, we could have chosen additional dimensions to test construct validity. Additionally, we only tested this scale with one wave of data and were not able to reassess item performance in a second wave. Lastly, this is a community level gender norms scale, so all statements start with, “in most families you know.” Other scales could measure family gender norms or norms within a specific religious community. We chose community norms to be broader and to measure one level consistently well.

Despite these limitations, our study has several strengths. Specifically, we used a large sample size to validate this scale. Scale validation claims that a sample size of ten is needed for each item, so with thirty items, we would need 300 women. Our sample of 2,445 far surpassed this minimum [44]. As is best practice, we used qualitative data from the community to inform the items and conducted cognitive testing to ensure that each item that we tested was understood as we had intended. Additionally, Ugandan researchers were closely involved in every aspect of the research, from data collection to item creation, item editing, data analysis, and manuscript review to ensure cultural relevance. Moreover, the G-NORM does not have one focus within gender norms (e.g., reproductive health decision making or women’s economic empowerment) but covers the complex phenomena of gender norms.

## Conclusion

Gender norms serve as a multi-faceted determinant of health and well-being and require measurement tools which account for their conceptual complexity. Validating the G-NORM in Uganda expands measurement options for researchers in the sub-Saharan African region working to change gender norms to reduce health inequalities or to understand the gender normative context before beginning a study. The two sub scales enable researchers to examine and identify which gender norms are more amenable to change, differ between descriptive and injunctive norms, and leverage this knowledge to shape targeted communication or behavior change interventions. Overall, the G-NORM provides a novel gender norms measure for researchers and interventionists working to improve women’s livelihood in sub-Saharan Africa.

## Supporting information

S1 File (DOCX)

S1 Questionnaire (DOCX)

## References

[1] Paudel Mohan, Javanparast Sara, Dasvarma Gouranga, and Newman Lareen. 2018. “A Qualitative Study about the Gendered Experiences of Motherhood and Perinatal Mortality in Mountain Villages of Nepal: Implications for Improving Perinatal Survival.” *BMC Pregnancy and Childbirth* 18 (1): 163. doi: 10.1186/s12884-018-1776-3 29764385 PMC5952814

[2] Singh Kavita, Haney Erica, and Olorunsaiye Comfort. 2013. “Maternal Autonomy and Attitudes Towards Gender Norms: Associations with Childhood Immunization in Nigeria.” *Maternal and Child Health Journal* 17 (5): 837–41. doi: 10.1007/s10995-012-1060-5 22696106 PMC3966061

[3] Cianelli Rosina, Ferrer Lilian, and McElmurry Beverly J. 2008. “HIV Prevention and Low‐income Chilean Women: Machismo, Marianismo and HIV Misconceptions.” *Culture*, *Health & Sexuality* 10 (3): 297–306. doi: 10.1080/13691050701861439 18432428 PMC2603075

[4] Brinton Mary C., and Lee Dong-Ju. 2016. “Gender-Role Ideology, Labor Market Institutions, and Post-Industrial Fertility: Gender-Role Ideology and Post-Industrial Fertility.” *Population and Development Review* 42 (3): 405–33. 10.1111/padr.161.

[5] Field Erica, Pande Rohini, Rigol Natalia, Schaner Simone, and Charity Troyer Moore. 2021. “On Her Own Account: How Strengthening Women’s Financial Control Impacts Labor Supply and Gender Norms.” *American Economic Review* 111 (7): 2342–75. doi: 10.1257/aer.20200705 37201159 PMC10191162

[6] Sedlander Erica, Dahal Minakshi, Jeffrey Bart Bingenheimer Mahesh C. Puri, Rimal Rajiv N., Granovsky Rachel, and Nadia G. Diamond‐Smith. 2023. “Adapting and Validating the G‐NORM (Gender Norms Scale) in Nepal: An Examination of How Gender Norms Are Associated with Agency and Reproductive Health Outcomes.” *Studies in Family Planning*, January, sifp.12231. doi: 10.1111/sifp.12231 36715570

[7] Newmann Sara J., Jennifer Monroe Zakaras Shari L. Dworkin, Withers Mellissa, Ndunyu Louisa, Gitome Serah, et al. 2021. “Measuring Men’s Gender Norm Beliefs Related to Contraception: Development of the Masculine Norms and Family Planning Acceptance Scale.” *Archives of Sexual Behavior* 50 (6): 2691–2702. doi: 10.1007/s10508-021-01941-w 33821378 PMC8416878

[8] Withers Mellissa, Dworkin Shari L., Onono Maricianah, Oyier Beryl, Cohen Craig R., Bukusi Elizabeth A., et al. 2015. “Men’s Perspectives on Their Role in Family Planning in Nyanza Province, Kenya.” *Studies in Family Planning* 46 (2): 201–15. doi: 10.1111/j.1728-4465.2015.00024.x 26059990

[9] Withers Mellissa, Dworkin Shari L., Zakaras Jennifer M., Onono Maricianah, Oyier Beryl, Cohen Craig R., et al. 2015. “‘Women Now Wear Trousers’: Men’s Perceptions of Family Planning in the Context of Changing Gender Relations in Western Kenya.” *Culture*, *Health & Sexuality* 17 (9): 1132–46. doi: 10.1080/13691058.2015.1043144 26032620

[10] Cislaghi Beniamino, and Heise Lori, 2020. “Gender Norms and Social Norms: Differences, Similarities and Why They Matter in Prevention Science.” *Sociology of Health & Illness* 42 (2): 407–22. doi: 10.1111/1467-9566.13008 31833073 PMC7028109

[11] Rimal Rajiv N., and Lapinski Maria K. 2015. “A Re-Explication of Social Norms, Ten Years Later: Social Norms.” *Communication Theory* 25 (4): 393–409. 10.1111/comt.12080.

[12] Miller DT, Prentice DA. Changing Norms to Change Behavior. *Annual Review of Psychology*. 2016;67(1):339–361. doi: 10.1146/annurev-psych-010814-015013 26253542

[13] Weber, Ann M, Beniamino Cislaghi, Valerie Meausoone, Safa Abdalla, Iván Mejía-Guevara, Loftus Pooja, et al. 2019. “Gender Norms and Health: Insights from Global Survey Data.” *The Lancet* 393 (10189): 2455–68. doi: 10.1016/S0140-6736(19)30765-2 31155273

[14] Learning Collaborative to Advance Normative Change. 2019. “Resources for Measuring Social Norms: A Practical Guide for Program Implementers.” Washington, DC: Institute for Reproductive Health, Georgetown University.

[15] Rimal Rajiv N., and Real Kevin. 2005. “How Behaviors Are Influenced by Perceived Norms: A Test of the Theory of Normative Social Behavior.” *Communication Research* 32 (3): 389–414. 10.1177/0093650205275385.

[16] Cislaghi Beniamino, and Heise Lori. 2018. “Theory and Practice of Social Norms Interventions: Eight Common Pitfalls.” *Globalization and Health* 14 (1): 83. doi: 10.1186/s12992-018-0398-x 30119638 PMC6098623

[17] Heise, Lori L, and Andreas Kotsadam. 2015. “Cross-National and Multilevel Correlates of Partner Violence: An Analysis of Data from Population-Based Surveys.” *The Lancet Global Health* 3 (6): e332–40. doi: 10.1016/S2214-109X(15)00013-3 26001577

[18] Center on Gender Health and Equity (GEH). (2020). A Roadmap for Measuring Agency and Social Norms in Women’s Economic Empowerment. n.d.

[19] Mackie G; Moneti F; Shakya H. 2015. “What Are Social Norms? How Are They Measured? 2015.” UNICEF/University of Califronia, San Diego, Center on Global Justice.

[20] Pulerwitz J., and Barker G. 2007. “Measuring Attitudes toward Gender Norms among Young Men in Brazil: Development and Psychometric Evaluation of the GEM Scale.” *Men and Masculinities* 10 (3): 322–38. 10.1177/1097184X06298778.

[21] Dempsey Robert C., McAlaney John, and Bewick Bridgette M. 2018. “A Critical Appraisal of the Social Norms Approach as an Interventional Strategy for Health-Related Behavior and Attitude Change.” *Frontiers in Psychology* 9. https://www.frontiersin.org/articles/10.3389/fpsyg.2018.02180. doi: 10.3389/fpsyg.2018.02180 30459694 PMC6232455

[22] Moreau C., Li M., De Meyer S., Loi Vu Manh G. Guiella R. Acharya, Bello B, Maina B, and Mmari K. 2019. “Measuring Gender Norms about Relationships in Early Adolescence: Results from the Global Early Adolescent Study.” *SSM—Population Health* 7 (April): 100314. doi: 10.1016/j.ssmph.2018.10.014 30581959 PMC6293033

[23] Baird Sarah, Bhutta Zulfiqar A., Bassam Abu Hamad Joan Hamory Hicks, Jones Nicola, and Muz Jennifer. 2019. “Do Restrictive Gender Attitudes and Norms Influence Physical and Mental Health during Very Young Adolescence? Evidence from Bangladesh and Ethiopia.” *SSM—Population Health* 9 (December): 100480. doi: 10.1016/j.ssmph.2019.100480 31993481 PMC6978471

[24] Costenbader Elizabeth, Zissette Seth, Martinez Andres, Katherine LeMasters Nana Apenem Dagadu, Deepan Prabu, et al. 2019. “Getting to Intent: Are Social Norms Influencing Intentions to Use Modern Contraception in the DRC?” Edited by Thach Duc Tran. *PLOS ONE* 14 (7): e0219617. 10.1371/journal.pone.0219617. 31310641 PMC6634398

[25] Sedlander Erica, Bingenheimer Jeffrey B., Long Michael W., Swain Minati, and Rimal Rajiv N. 2022. “The G-NORM Scale: Development and Validation of a Theory-Based Gender Norms Scale.” *Sex Roles* 87 (5–6): 350–63. doi: 10.1007/s11199-022-01319-9 36168556 PMC9508194

[26] Seguino S. PlusÇa Change?1 evidence on global trends in gender norms and stereotypes. *Feminist Economics*. 2007;13(2):1–28. doi: 10.1080/13545700601184880

[27] Rosenberg and Edmeades. 2023. “Trends in Men’s Gender Attitudes: Progress, Backsliding, or Stagnation?” Rockville, Maryland, USA: ICF.

[28] Mulawa Marta I., H. Luz McNaughton Reyes, Vangie A. Foshee, Carolyn T. Halpern, Sandra L. Martin, Lusajo J. Kajula, et al. 2018. “Associations Between Peer Network Gender Norms and the Perpetration of Intimate Partner Violence Among Urban Tanzanian Men: A Multilevel Analysis.” *Prevention Science* 19 (4): 427–36. doi: 10.1007/s11121-017-0835-8 28849338 PMC5832502

[29] Uganda Family Planning Atlas. USAID, April, 2019.

[30] Suchman Lauren, Omoluabi Elizabeth, Kramer Julia, Vallin Janelli, Sedlander Erica, Gitome Serah, et al. 2023. “Analyzing Fast and Slow: Combining Traditional and Rapid Qualitative Analysis to Meet Multiple Objectives of a Complex Transnational Study.” *Frontiers in Sociology* 8 (February): 961202. doi: 10.3389/fsoc.2023.961202 36818663 PMC9931144

[31] Connell Raewyn. 2013. *Gender and Power*: *Society*, *the Person and Sexual Politics*. John Wiley & Sons.

[32] Wingood Gina M., and DiClemente Ralph J. 2000. “Application of the Theory of Gender and Power to Examine HIV-Related Exposures, Risk Factors, and Effective Interventions for Women.” *Health Education & Behavior* 27 (5): 539–65. doi: 10.1177/109019810002700502 11009126

[33] DeVellis R. F., and Thorpe C.T. 2021. *Scale Development*: *Theory and Application 5th Ed*. Sage Publications.

[34] Vu Lung, Pulerwitz Julie, Brady Burnett-Zieman Cecily Banura, Okal Jerry, and Yam Eileen. 2017. “Inequitable Gender Norms From Early Adolescence to Young Adulthood in Uganda: Tool Validation and Differences Across Age Groups.” *Journal of Adolescent Health* 60 (2): S15–21. 10.1016/j.jadohealth.2016.09.027. 28109335

[35] Comrey A, Lee H. A. 1992. *First Course in Factor Analysis*. Hillsdale, NJ.

[36] Vandenberg RJ, Lance CE. A Review and Synthesis of the Measurement Invariance Literature: Suggestions, Practices, and Recommendations for Organizational Research. Organizational Research Methods. 2000;3(1):4–70. doi: 10.1177/109442810031002

[37] Mosha Idda, Ruben Ruerd, and Kakoko Deodatus. 2013. “Family Planning Decisions, Perceptions and Gender Dynamics among Couples in Mwanza, Tanzania: A Qualitative Study.” *BMC Public Health* 13: 523. doi: 10.1186/1471-2458-13-523 23721196 PMC3679800

[38] Fleming Paul J., Silverman Jay, Ghule Mohan, Ritter Julie, Battala Madhusudana, Velhal Gajanan, et al. 2018. “Can a Gender Equity and Family Planning Intervention for Men Change Their Gender Ideology? Results from the CHARM Intervention in Rural India: Can a Gender Equity and Family Planning Intervention for Men Change Their Gender Ideology?” *Studies in Family Planning* 49 (1): 41–56. 10.1111/sifp.12047. 29441577 PMC6469641

[39] Fitriana Nina, Fonny Dameaty Hutagalung Zainudin Awang, and Sumaia Mohammed Zaid. 2022. “Happiness at Work: A Cross-Cultural Validation of Happiness at Work Scale.” Edited by Michael B. Steinborn. *PLOS ONE* 17 (1): e0261617. 10.1371/journal.pone.0261617. 34986180 PMC8730434

[40] Zimmerman Linnea A., Koenig Leah R., Pulerwitz Julie, Kayembe Patrick, Maddeleno Matilde, and Moreau Caroline. 2021. “The Intersection of Power and Gender: Examining the Relationship of Empowerment and Gender-Unequal Norms Among Young Adolescents in Kinshasa, DRC.” *Journal of Adolescent Health* 69 (1): S64–71. doi: 10.1016/j.jadohealth.2021.03.031 34217462

[41] Holt K, Challa S, Alitubeera P, et al. Conceptualizing Contraceptive Agency: A Critical Step to Enable Human Rights-Based Family Planning Programs and Measurement. Glob Health Sci Pract. Published online February 12, 2024:ghsp;GHSP-D-23-00299v1. doi: 10.9745/GHSP-D-23-00299 38346841 PMC10906552

[42] Holt K, Galavotti C, Omoluabi E, Challa S, Waiswa P, Liu J. Preference‐Aligned Fertility Management as a Person‐Centered Alternative to Contraceptive Use‐Focused Measures. Studies in Family Planning. 2023;54(1):301–308. doi: 10.1111/sifp.12228 36723038

[43] Harper C.C., Rao L., Muñoz I. et al. Agency in Contraceptive Decision-Making in Patient Care: a Psychometric Measure. J GEN INTERN MED 38, 1366–1374 (2023). doi: 10.1007/s11606-022-07774-0 36070169 PMC10160288

[44] Nunnaly JC. 1978. *Psychometric Theory*. New York: McGraw-Hill.

