# Supplementary material for: Adaptation of the G-NORM (Gender norms scale) in Uganda: An examination of how gender norms are associated with reproductive health decision-making
Source: PLoS One. 2024 Nov 4;19(11):e0308249. doi: 10.1371/journal.pone.0308249 (PMC11534242; doi:10.1371/journal.pone.0308249)
Supplement: S1 File — (DOCX) [file pone.0308249.s001.docx]

*Uganda G-NORM Scale (20 items total – ten items in each sub-scale)*

| **Descriptive norms** |
| --- |
| *In most families you know if a woman earns money, it will cause problems in her marriage. |
| In most families you know only men make decisions about household income and expenses. |
| In most families you know husbands make the final decision about how many children to have. |
| In most families you know men make the final decision about their wife (or partner) using family planning methods. |
| *In most families you know if a woman disobeys her husband, she is sent back to her parents (or sent away). |
| In most families you know only women do the cooking, cleaning, and caring of children. |
| In most families you know women stop working when they get married. |
| In most families you know girls stop going to school if they get pregnant. |
| In most families you know husbands make the final decisions about buying major household items (e.g., television, bicycle, cell phone) |
| In most families you know if there is only enough money for one cell phone for the household, the husband owns it. |
|  |
| **Injunctive norms** |
| Most families you know believe that a woman should not work outside the home to keep peace in her marriage. |
| Most families you know believe that only men should make decisions about income and expenses. |
| Most families you know believe that husbands should make the final decision about how many children to have. |
| Most families you know believe that men should make the final decision about their wife using family planning |
| Most families you know believe that if a woman disobeys her husband, she should be sent back to her parents (or sent away). |
| Most families you know believe that only women should do the cooking, cleaning, and caring of children. |
| Most families you know believe that women should stop working when they get married. |
| Most families you know believe that girls should stop going to school if they get pregnant. |
| Most families you know believe that husbands should make the final decisions about buying major household items (e.g., television, bicycle, cell phone). |
| Most families you know believe that if there is only enough money for one cell phone for the household, the husband should own it. |

*All response options are on a 4-point likert scale: strongly disagree, disagree, agree, strongly agree

*Uganda specific items are highlighted in grey *items that indicate social sanctions (i.e., repercussions if someone breaks a social norm) have an asterik next to them
